# Engineering serendipity: High-throughput discovery of materials that resist bacterial attachment^☆^

**DOI:** 10.1016/j.actbio.2015.11.008

**Published:** 2016-04-01

**Authors:** E.P. Magennis, A.L. Hook, M.C. Davies, C. Alexander, P. Williams, M.R. Alexander

**Affiliations:** a Laboratory of Biophysics and Surface Analysis, School of Pharmacy, University of Nottingham, Nottingham, UK; b Drug Delivery and Tissue Engineering, School of Pharmacy, University of Nottingham, Nottingham, UK; c School of Molecular Medical Sciences, University of Nottingham, Nottingham, UK

**Keywords:** Biomaterials, Bacteria, High-throughput, Biofilm, Polymers

## Abstract

Controlling the colonisation of materials by microorganisms is important in a wide range of industries and clinical settings. To date, the underlying mechanisms that govern the interactions of bacteria with material surfaces remain poorly understood, limiting the *ab initio* design and engineering of biomaterials to control bacterial attachment. Combinatorial approaches involving high-throughput screening have emerged as key tools for identifying materials to control bacterial attachment. The hundreds of different materials assessed using these methods can be carried out with the aid of computational modelling. This approach can develop an understanding of the rules used to predict bacterial attachment to surfaces of non-toxic synthetic materials. Here we outline our view on the state of this field and the challenges and opportunities in this area for the coming years.

**Statement of significance:**

This opinion article on high throughput screening methods reflects one aspect of how the field of biomaterials research has developed and progressed. The piece takes the reader through key developments in biomaterials discovery, particularly focusing on need to reduce bacterial colonisation of surfaces. Such bacterial resistant surfaces are increasingly required in this age of antibiotic resistance. The influence and origin of high-throughput methods are discussed with insights into the future of biomaterials development where computational methods may drive materials development into new fertile areas of discovery.

New biomaterials will exhibit responsiveness to adapt to the biological environment and promote better integration and reduced rejection or infection.

## Graphical abstract

**Figure:**
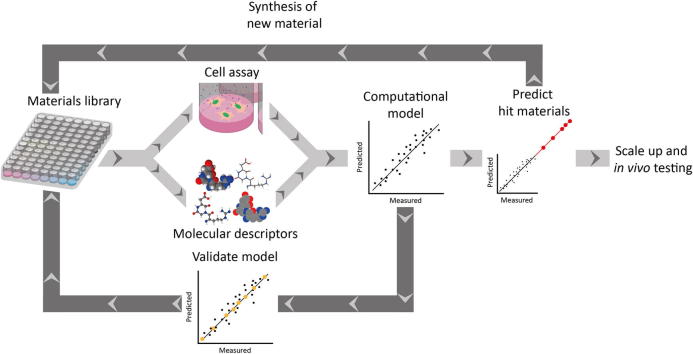

## 1 The bacterial challenge

Antimicrobial resistance has been predicted to rival cancer as both a cause of death and an expense to healthcare systems by 2050 [1]. Bacteria inflict significant human suffering through acute and chronic disease. Infection has serious impacts upon morbidity and mortality [2], [3]. Socio-economic factors associated with infectious diseases have negative influences upon trade, commerce and social development [4], [5]. Infectious diseases are problematic for both patients and society as a whole, for example the cost of *Clostridium difficile* infections in the US alone is estimated at over $796 million [6] and the total cost for healthcare-associated infections (HAI) is between $28 billion and $45 billion per year [7].

A recently published prevalence survey identified that in 2011 medical-device associated infections accounted for 25.6% of healthcare associated infections [8]. Many types of devices, such as venous catheters and prosthetic heart valves, become colonised by bacteria which can subsequently form biofilms and cause infection and device failure [9]. In 2009 it was estimated that in the US alone there are a quarter of a million central-line-associated bloodstream infections annually leading to 31,000 deaths per year [7]. The treatment of device-associated infections often proves particularly challenging as micro-organisms within a biofilm are able to protect themselves from the immune system and antibiotics [10]. Bacteria are estimated to be 10–1000 times more tolerant to host defences and antibiotics than in their planktonic state [11], [12]. For example the bactericidal concentration of a particular systemic dose of vancomycin for *Staphylococcus epidermidis* increases from 6.25 micrograms/mL to 400 micrograms/mL when the bacterium moves from the planktonic state to form a biofilm [13], [14], [15], [16]. Following years of repeated and prolonged use and mis-use of antibiotics, anti-microbial resistance is now a global threat [17]. A preferred approach is therefore to avoid the use of antibiotics and biocidal agents and to prevent the development of device associated infections by preventing surface colonisation and biofilm formation.

The first stage of biofilm formation involves the initial attachment of individual bacterial cells or small bacterial aggregates, which is usually preceded by adsorption of biological macromolecules [11]. Systems designed to prevent bacterial attachment aim to disrupt biofilms at the earliest possible stage. Polymer materials are well suited to biofilm prevention. It has been shown that they can be readily tailored by variation in their chemistry to achieve non-fouling effects [22]. One problem is that the required chemistry cannot readily be predicted from first principles.

The approaches employed to resist biofilm formation are either the production of cytotoxic materials designed to kill bacteria upon contact [18], [19], [20], which are unlikely to select for resistance [21], or anti-adhesion strategies whereby the materials circumvent bacterial attachment, biofilm formation and the hence associated increase in resistance to antibiotics and host defences. Compared to antibiotic containing materials, surfaces that resist bacterial attachment do not induce the evolutionary pressure which would lead to bacterial resistance. This characteristic means that this class of material is of particular interest in an age of growing antibiotic resistance. A number of anti-fouling polymer strategies have recently been reviewed by Rosenhahn et al. [22]. The mechanisms that have been employed to prevent attachment include electrostatic repulsion, steric repulsion, topography and hydration.

Kosmotropes, which stabilise proteins in their native form, particularly poly(ethylene glycol) and zwitterionic polymers have attracted the most research attention as anti-fouling films for their ability to prevent cell attachment [23]. Their discovery through early observations of their resistance to coating by proteins have now led to wide spread use [24].

Poly(ethylene glycol) acts by hydrogen bonding to up to three water molecules per repeating ether group. Through this mechanism, the complex is sterically stabilised. In order for protein to adsorb the chains must be compressed and water released. The removal of water from the chains has an enthalpic cost. The compression of the chains has a corresponding entropic expense [25], [26], [27], [28]. However hydrophilicity and steric hinderance alone do not explain its efficiency suggesting its unique solution properties contribute to its non-fouling ability [29]. Zwitterionic polymers are electrically neutral yet tightly co-ordinate water through ionic interactions. This results in a highly hydrophilic surface [30] where, similarly, the removal of water is entropically unfavourable [22].

However, protein adhesion is a process which one can reasonably assume may be described by physicochemical processes. It is well known that mammalian cellular adhesion is regulated by protein adsorption through integrin–peptide interactions [31]. There have been attempts to use physicochemical parameters to explain bacterial attachment [21], [32], [33]. Recently superwettable materials have gained interest to aid understanding of bacterial interactions with certain materials. Bacterial surface components such as peptidoglycan have shown to influence adherence with surfaces of varying hydrophilicity [34]. However predicting attachment across our wide chemical libraries using water contact angle has not been successful, apart from in very limited subsets of similar materials, leading us to conclude that the use of the wettability as a surface descriptor (hydrophobic or hydrophilic) is not helpful in understanding bacterial–surface interactions [35]. This points to the importance of sophisticated bacterial sensing mechanisms and downstream cellular responses in determining their responses to a specific material. Bacterial cells do not behave as inanimate objects but possess complex regulatory network systems for sensing and mechanics, for example type IV pili [25], [29], [36] and other fimbrial types [37] that help determine their reactions to different surfaces. This flexibility presents a difficult problem to identify attachment resistance surfaces.

Despite significant on-going research into anti-adhesive surfaces there has been a lack of translation from successful laboratory-based systems to clinically useful medical devices, many of which still employ high-fouling surfaces. Some instances where translation has been successful include the PolySB coating based upon work by Loose et al. at Massachusetts Institute of Technology [38] and Avert™, a poly(ethylene glycol) and biguanide coating produced by Biointeractions Limited [39]. Using an drug eluting system, J. Kohn developed TYRX™ which employs a polymer discovered in a high throughput screening campaign. The product is constructed of a polymer, containing rifampicin and minocycline which may be absorbable or non-absorbable [40]. A retrospective, observational analysis demonstrated the coating to reduce the risk of infections and patient mortality [41].

Busscher et al. discussed the issues facing translation of outcomes from scientific studies into successful biomedical devices. The delicate interplay between bacteria and host cells, such as epithelial cells, that leads to colonisation of an implanted surface has, thus far, been difficult to replicate in laboratory studies. Improving these *ex vivo* models should help improve translation to the clinic [42]. These experimental issues are compounded by the number of patients and the duration required for clinical trials to demonstrate efficacy and safety unequivocally [43]. Beyond innovation barriers, new approaches are required between academic discovery groups and those in industrial and regulatory areas to increase translation to improve health outcomes [9]. For example, in academia there is a need to publish and this can often be preceded by inadequate patenting or none at all. They believe that this lack of intellectual property demotivates industrial players from developing new ideas which could drive further translation.

## 2 Biomaterials development

Existing biomedical materials have arisen from a combination of accessibility and utility. For example silicone rubber, originally developed as an electrical insulator, finds common application as a medical device material due to its useful mechanical properties and inert nature. Polymethacrylates originally used in the plastic canopies on warplanes were subsequently exploited for use in intraocular lenses since it was noted that polymer fragments trapped in pilots’ eyes were well tolerated [44]. Whilst the mechanical and other bulk properties may be optimal for these materials for a given application, the tendency to promote bacterial attachment and biofilm formation is not. Consequently, modification of these basic materials offers one route to new biomaterials which has been explored, e.g. copolymerisation and surface functionalisation. However, an alternative is to start afresh and ask the question, what would the optimal material be for a particular application? The aim of this article is to guide the reader through steps taken thus far to control bacterial biomaterial interactions and how this area could develop in a different but exciting direction by answering this question.

Over the last decade, dramatic advances have been made through both hypotheses relating material properties to cellular responses, and discovery of new materials made using high-throughput screening [45], [46], [47], [48], [49]. Useful illustrations of the step change jumps in knowledge are the material property-cell differentiation relationships characterised for mesenchymal cells. These include Engler and Discher’s data that substrate stiffness could direct stem cell fate [50], Dalby and Oreffo’s observation that nanotopography could control cell differentiation [51] and Anseth’s correlation of chemistry in 3D cell encapsulating gels with differentiation lineage [52]. However the relative contributions of these effects on cell fate are poorly defined and similar relationships have not been established for microbial cells.

Despite these advances, rational design of new biomaterials is still hindered by the paucity of information on the physicochemical parameters governing the response of different cell types of interest to a broad range of materials.

### 2.1 High-throughput approaches

The rational design roadblock for biomaterials has promoted an interest in the application of data driven, high-throughput screening approaches that can be applied to any cell type and adapted to model a particular application area/service environment [53]. Mounting screening campaigns with large material libraries could be referred to as engineering “*happy accidents*”, or serendipity. This was first illustrated in 2004 for polymer micro arrays by Anderson et al. [55] for stem cells and also exemplified by the Bradley group [56]. In addition to the identification of hit materials for further development towards materials for cell control, the large amount of information generated on the biointerface can be used to obtain new insights. To achieve structure–property relationships requires analysis of the surface chemistry rather than assumption of its identity from the input monomers, which is where the development of high-throughput surface characterisation has been critical [57], [58]. This has facilitated correlations of the surface chemistry and monomer identity with bacterial attachment to move towards rational design [59], [60], combining an understanding of both the organism and materials to propose new biomaterials *ab initio* [61].

The success of the data driven scientific method illustrated using the high-throughput screening approach demonstrates the validity of its application for materials discovery. Recently, Autefage et al. used a non-reductionist approach to understand the influence of strontium ion incorporation into 45S5 bioactive glass. Their unbiased investigation found a number of changes, including increases in cellular and membrane cholesterol content and in phosphorylated myosin II light chain which may not have be identified from biological predictions alone [65]. This contrasts with reductionist approaches favourably, indicating the need to identify new materials but then understand their mode of action through hypothesis driven investigation [54].

Brocchini et al. in 1998 applied the combinatorial materials screening approach to polymeric biomaterials discovery. The authors synthesised separately but in parallel, 112 biodegradable polyacrylates from 8 aliphatic diacids and 14 diphenols before subsequent surface coating and testing [69]. Significantly, the authors discovered that while increasing the surface wettability improved fibroblast growth, backbone substitution with an oxygen also improved growth without affecting surface hydrophilicity. This showed that single characteristics such as water contact angle (WCA) cannot be used to guide material development for complex living systems. The procedure allowed many materials and their characteristics to be analysed, however, the separate syntheses and subsequent coating made the process unacceptably slow for ultra high-throughput novel materials discovery where rapid evolution from one generation to the next in response to cell response data is desirable.

**Fig. 1:**
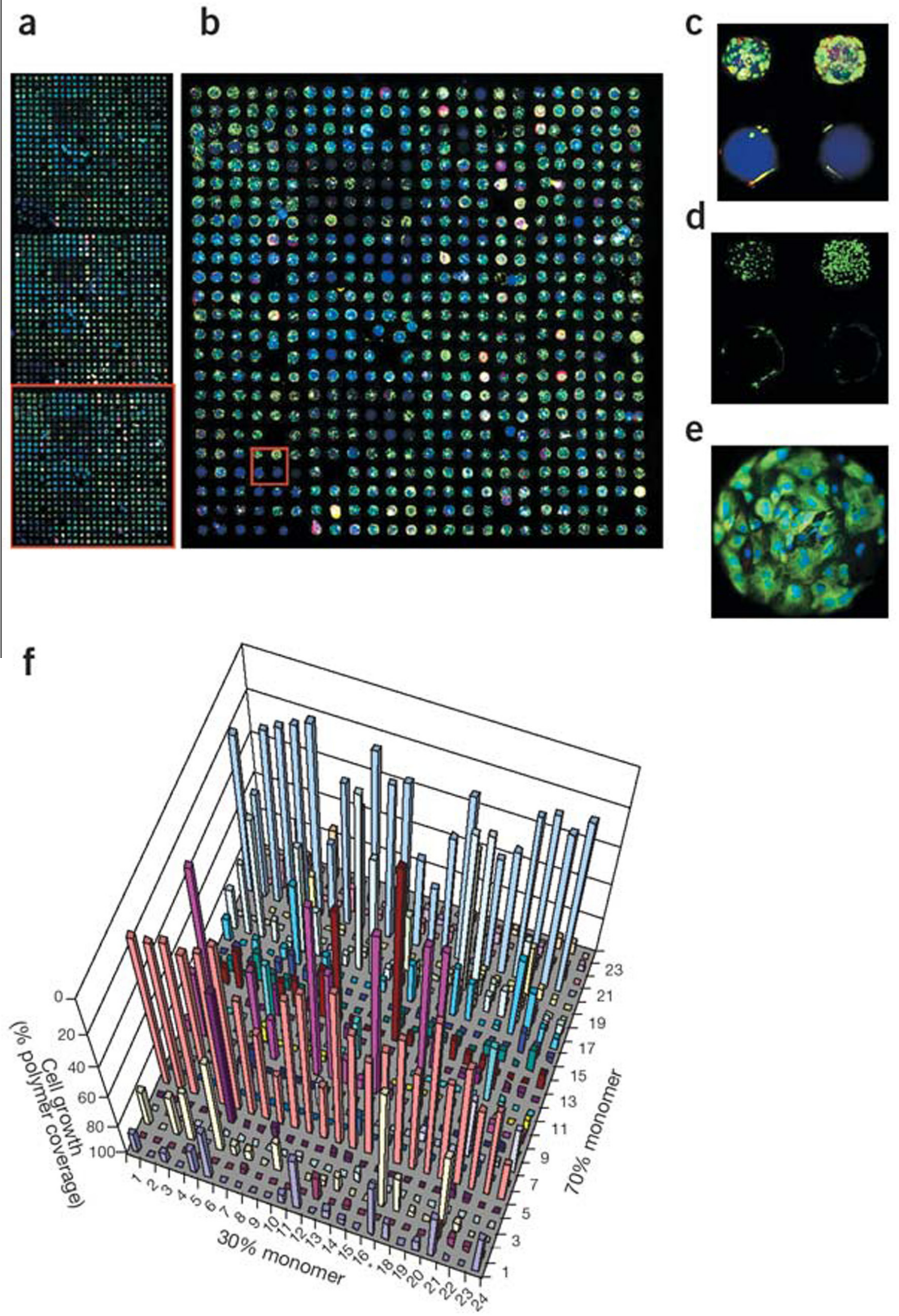
(a–c) Six million human embryonic stem (hES) cell embryoid body day-6 cells were inoculated on the polymer array in the presence of retinoic acid for 6 d and then stained for cytokeratin 7 (green) and vimentin (red). Polymer spots can be identified by blue fluorescence. (d) Nuclei were also stained (green) (not shown in other images to simplify presentation). (e) Typical cytokeratin 7 positive spot on polymer composed of 100% monomer 9. (f) Cell attachment and growth as a function of polymer composition, measured as the average percent cell coverage of a polymer spot on day 6. Although the vast majority of polymers remained attached to the matrix during analysis, certain hydrophilic polymers (such as those composed of 30% monomer *) did fall off after extensive submersion [55].

In order to optimise the rate at which new biomaterials could be discovered and their biological properties assessed, the microarray format has now become routine. In this way, hundreds of unique polymers are generated on-slide and assayed on a single substrate in a single experiment. The group of Langer et al. first published the use of microarrays to screen hundreds of materials for their effects upon the growth and differentiation of human stem cells [55]. This initial report described 576 unique polymers in triplicate, generated *in situ* by printing monomers pairwise into an array and curing using UV on 25 × 75 mm pHEMA coated glass slides (Fig. 1). Commercial monomers were employed to enable ready access to a large chemical space.

This enabled rapid, simultaneous assays to be carried out in parallel. The new platform set the precedent for high-throughput material discovery for the purposes of controlling cell attachment and growth including bacteria [48], [49]. The different methods which can be used to prepare microarrays have been reviewed recently in the literature [70].

Subsequently, there have been a number of notable successes for the high-throughput screening approach for discovery of novel biomaterials including the identification of a new class of polymers resistant to bacterial attachment that has potential as medical device coatings [71]. More recently, also a series of materials that allow long term renewal of pluripotent stem cells [46]. These finds have been achieved using unbiased screening or “*fishing expeditions*” a commonly used pejorative description of this approach.

**Fig. 2:**
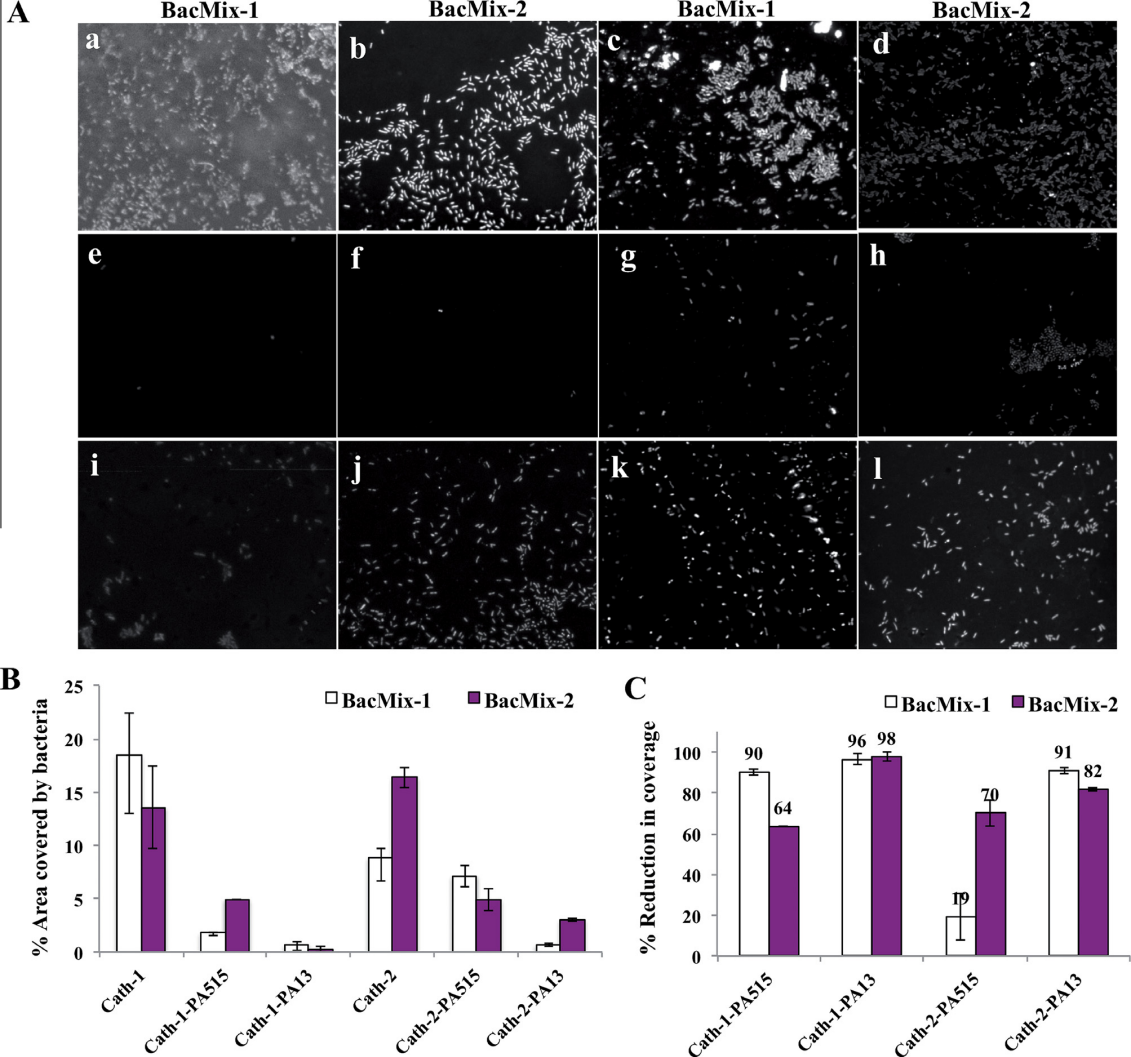
(A) Confocal fluorescence images (×40 magnification, *λ*ex 405 nm, *λ*em 414–502 nm) of catheter surfaces showing bacteria binding/non-binding. The images were obtained after 72 h incubation with bacteria, followed by washing, fixing and staining with DAPI (1 μg mL^−1^). (a and b) Uncoated Cath-1, (c and d) uncoated Cath-2, (e and f) Cath-1-PA13, (g and h) Cath-2-PA13, (i and j) Cath-1-PA515, (k and l) Cath-2-PA515. (B) The percentage of surface area covered by bacteria after 72 h incubation with BacMix-1 and BacMix-2, and (C) percentage of reduction in bacterial binding, obtained by comparing the area of bacterial coverage to the area of the image. PA13 Co-polymer of methylmethacrylate and dimethylacrylamide (9:1 monomer ratio) PA515 Co-polymer of methoxyethylmethacrylate, diethylaminoethylacrylate and methylmethacrylate (6:1:3 monomer ratio) [73].

When considering materials from which to manufacture medical devices with reduced rates of infections, the question is, are there materials better at resisting attachment of bacteria than poly(ethylene glycol) and zwitterionic polymers? The extension of the high-throughput platform was a logical transition to emulate the successes seen with eukaryotes. Pernagallo et al. produced an early report of an array to identify specific bacteria binding/non-binding polymers. The authors used clinically relevant strains and identified polyacrylate materials which had properties of high binding, low binding and selective binding [72]. The Bradley group later screened a 381 polymer library with up to eleven different bacterial strains/species including those obtained from endotracheal tubes or from patients with infectious endocarditis (BacMix-1 and BacMix-2). Further to their high-throughput screen a number of “hit” polyurethane and polyacrylates/acrylamides were identified. The hit coating, which was a co-polymer of methylmethacrylate and dimethylacrylamide in a molar ratio of 9:1 (PA13), generated ⩾96% reduction for the bacteria mixtures tested using a microaerobic environment and supplementing growth with BHI after 24 and 48 h (Fig. 2) [73].

The surface characteristics and properties of materials obtained from combinatorial methods cannot be entirely rationalised based upon the composition assumed from the raw materials used for synthesis, and as such surface analysis is a key component for understanding the materials’ behaviour [60]. To understand, explain and develop a biological observation from novel materials, the surface characteristics need to be described [70]. Traditional polymer characterisation methods do not generally provide information on the upper-most surface which controls cellular response to materials. This is problematic as importantly it is the surfaces of the materials which dictate the observed phenomena. To circumvent this analytics barrier, surface analysis techniques such as time of flight secondary ion mass spectroscopy (ToF-SIMS) [57], [58], atomic force microscopy (AFM) [74], surface wettability measured through water contact angles (WCA) [75], surface plasmon resonance (SPR) [76] and X-ray photoelectron spectroscopy (XPS) [58] allow for rapid characterisation of polymer microarrays. Together with the microarray format, these techniques are known as high-throughput surface characterisation (HTSC) [77].

The polymer array and combinatorial approach can be used to perform a biased screen, when the identity of the members of the library on the array are chosen in order to test preconceived notions of the function of certain monomer or monomer combinations when combined to make polymers. Alternatively an unbiased screen can be performed where the aim is to cover as wide a chemical space as possible in the hope that unexpected relationships may be discovered. This most exciting screening method provides the opportunity to discover new and unexpected materials and materials classes. After the response to a surface has been described, the nature of any interconnectiveness between the surface analysis data and biological response can be explored. ToF-SIMS surface analysis data is complex and multivariate. In order to overcome the challenge of information overload a mathematical technique called Partial Least Squares (PLS) has been applied. This is done to correlate multivariate datasets such as surface analysis to univariate data, for example the observed biological response [78]. This was successfully used to correlate the attachment of human embryoid body cells with the surface chemistry of the materials as measured by ToF-SIMS [77].

**Fig. 3:**
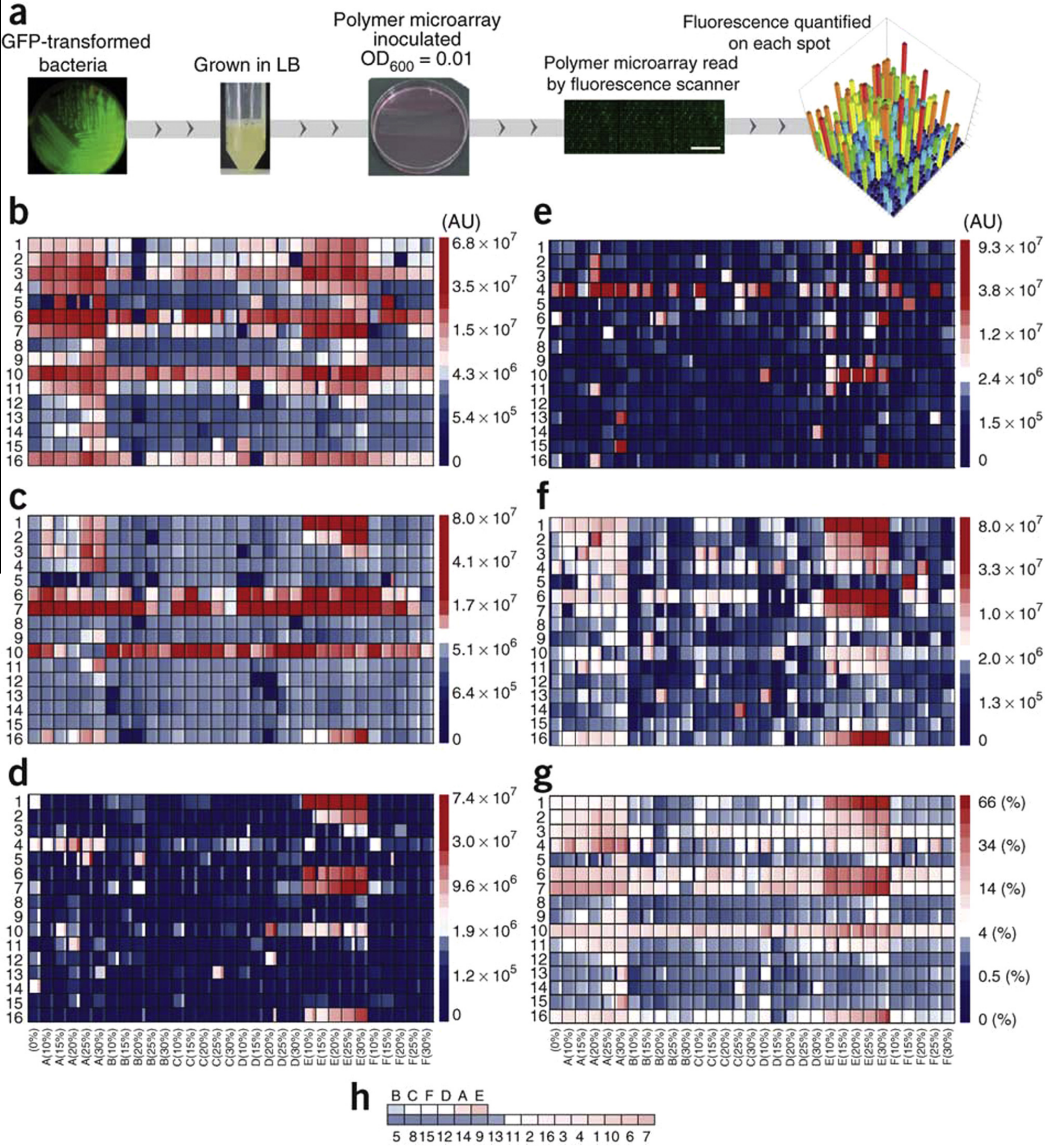
(a) Bacterial attachment assay procedure; (b–f) Intensity maps of F measured for each bacterial strain on the first-generation array after 72-h incubation; *Pseudomonas aeruginosa* (b), *Staphylococcus aureus* (c), Uropathogenic *Escherichia coli* (UPEC) (d), UPEC grown in artificial urine (e) and UPEC grown on an artificial urine-conditioned slide (f). (g) Intensity map of the bacterial performance obtained for each material in the array, given as a percentage of the maximum bacterial numbers for all strains under all culture conditions. The major monomers are listed on the *y*-axis whereas the composition of the minor monomers is shown on the *x*-axis. The large shaded area within each outlined area indicates the mean value, and the mean ± 1 s.d. unit is presented in the narrow columns to the right (plus) and left (minus) of the mean, *n* = 3. Key to right. A square-root scale is applied to the intensity indicators. (h) The average normalised fluorescent intensity for all materials containing a specific monomer, ranked from lowest to highest. The major and minor monomers were considered separately. The colour next to each monomer is indicative of that monomer’s mean ι and is coloured by the same intensity scale as in g [49].

The ToF-SIMS technique is versatile and has been used to identify key spectral ions responsible for reduced or increased bacterial attachment to a library of polymers [49]. Hook et al. generated an expansive combinatorial polymer microarray from 22 commercially available acrylate monomers. These were combined in varying ratios to create 496 unique polymers obtained through photo-initiated free-radical polymerisation. The array was incubated with fluorescent bacteria for three days and the resulting attachment or resistance to bacteria was quantified from the fluorescent signal (ι) on each polymer surface (Fig. 3).

The observed fluorescent signal could be predicted using linear PLS correlation for the bacteria *Pseudomonas aeruginosa* and *Staphylococcus aureus*. Cyclic hydrocarbons, esters, tertiary butyl moieties and non-aromatic hydrocarbons were identified from the ToF-SIMS signals as key to reduce bacterial attachment. This contrasted with ethylene glycol and hydroxyl groups that were found to correlate with higher bacterial attachment. These insights were used for the selection of ‘hit’ monomers to be used in a subsequent generation array that screened copolymer series for formulation optimisation. Furthermore, the chemical fragments associated with low bacterial attachment provided insight into the physio-chemical mechanism by which the polymers resisted bacteria. Specifically, the association of both the hydrophilic ester groups and hydrophobic cyclic hydrocarbon groups with low bacterial attachment suggested that the weakly amphiphilic nature of the polymers was key to their function. The scaled up material showed a thirty-fold reduction in biofilm formation compared to commercial antibacterial silver hydrogel. In the group’s later work, the microarray was expanded to generate 1273 individual polymers and clinically isolated pathogenic bacterial strains were included in the screen. From this larger screen, a greater number of materials had their antibacterial properties investigated. Lead hit materials showed up to 99% fewer bacteria attached compared to an antibacterial silver hydrogel [48].

### 2.2 Materiomic approaches

The number of potential polymers that could be synthesised are innumerable, thus, it is not experimentally feasible to screen all possible materials even utilising high-throughput screening methods. To truly assess the interaction of bacteria with the polymeric materials space another approach is required. The use of materiomics, involving computational and experimental approaches with large datasets for biomaterial discovery has recently been reviewed in a number of books and reviews [61], [79], [80].

Computational methods including quantitative structure–property relationships (QSPR) [81] are set to guide experimentation to potentially fertile new areas of chemical space using surface structure-performance relationships from both experimental molecular descriptor – bacterial response relationships and surface analytical – bacterial response data sets. Hypotheses can be formed and tested using cellular attachment information and the ToF-SIMS data to produce descriptors that can guide improved material development and resistance to bacterial attachment and colonisation. For a limited set of polymers, Sanni et al. developed a molecular descriptor parameter *α* (Eq. 1) that was found to correlate with bacterial cell resistance [35].

(1) α=0.44nRoTB-clogP

**Fig. 4:**
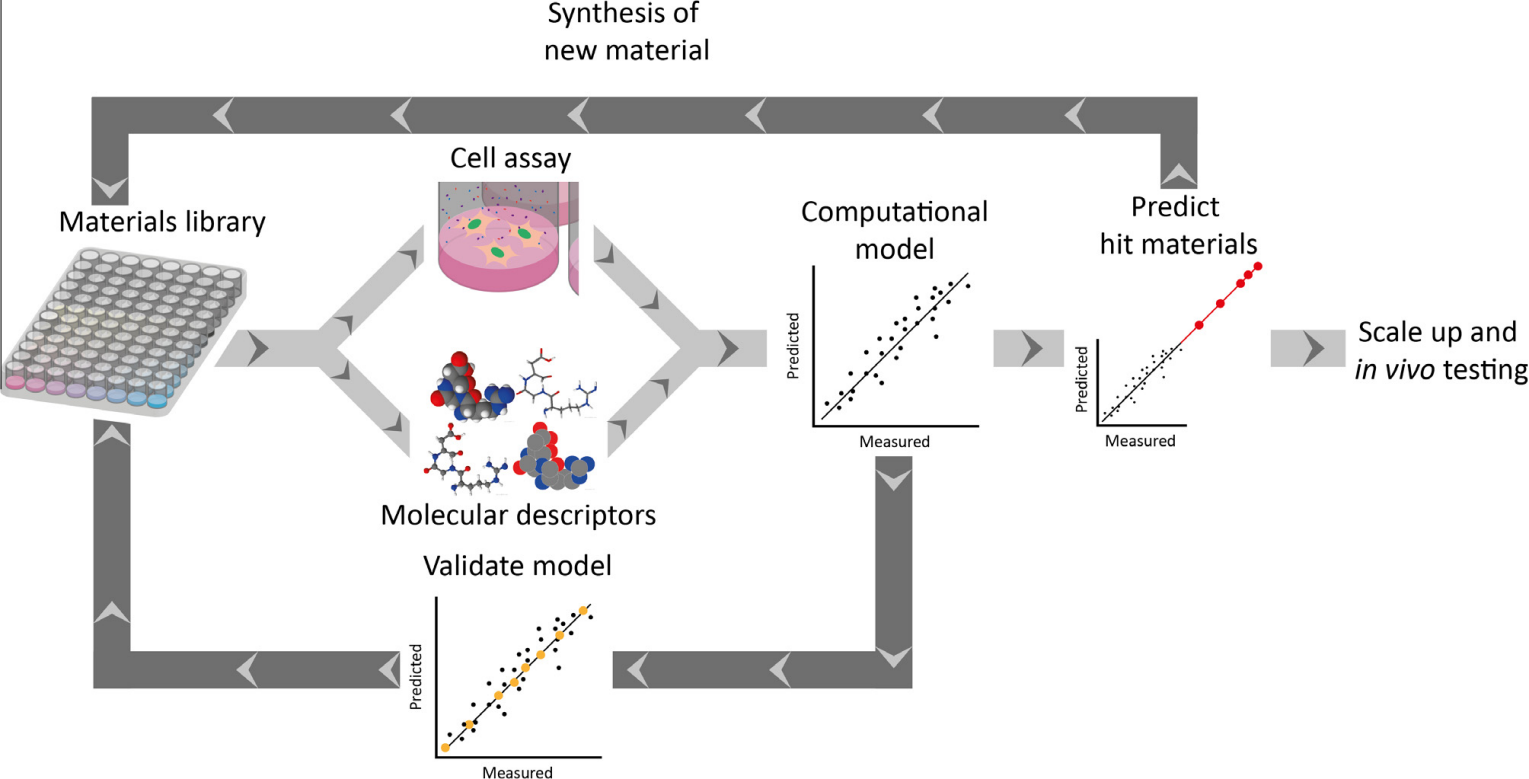
Process of computational modelling for the generation of novel biomaterials. Materials are screened from a library of materials and cell responses are modelled against molecular descriptors. The model is then used to predict new untested materials which are to be synthesised and tested. Optimised materials are then scaled up for in vivo testing.

This equation links chemical parameters of a biomaterial by the number of rotational bonds (*n*RotB) and hydrophobicity (*c*log*P*) to bacterial attachment on that surface. The challenge in this area, as it was for drug discovery, is to project outside the training set, to identify candidates outside the modelled materials space (Fig. 4).

The complex data processing requires machine learning systems capable of complex pattern recognition [82] beyond that achieved by PLS modelling [60]. Epa et al. in 2014 utilised a machine learning modelling approach to predict the attachment of *P. aeruginosa*, *S. aureus* and *Escherichia coli* to polymer surfaces from a library of computationally derived molecular descriptors [59]. Following predictions of attachment, several monomers that had not been used to generate the models were tested for their propensity for colonisation by *P. aeruginosa*. The model successfully predicted those monomers that would produce high and low adherent surfaces. A number of molecular descriptors were invoked to explain the bacterial attachment to the materials, including those incorporating the number and type of chemical functional groups in the polymers, descriptors relating to the ability of the polymers to form hydrogen bonds (relevant to biofilm formation), the dipole moments, surface wettability, molecular shape, and complexity. The successful utility of these descriptors to predict the biological performance of the materials suggests these properties play a key role in determining how bacteria attach or do not attach. As such, predictive models are useful not only as a materials development tool but also at providing novel insights into the underlying biological–material interactions. The ultimate goal is for modelling techniques to allow us to “*dial up*” biomaterials and drugs based upon the desired characteristics while minimising experimental time. Although this is far from where we are currently with biomaterials, one can look to the *in silico* design of aerospace components for inspiration.

## 3 Future outlook

Polymers offer many advantages as a material platform for manipulating bacteria–biomaterial interactions. Polyvalency and intricate property manipulation can allow for the development of precision surfaces and materials. The ability to produce polymers in a variety of formats, for example as three dimensional networks, soluble agents and surface coatings, allows for their application to a variety of analytic formats and uses.

Understanding the interactions of bacteria with biomaterials may be a route to improve functionality. Such intelligence-led approaches have been demonstrated and are guided by knowledge of organisms and their attachment processes [36], [37], [65]. However, the colonisation process, for example, is complex and not all steps are known and understood.

However, the future goal is not simply to prevent bacterial attachment but also to control the specific biological responses to a surface. Research is already underway into bioinspired devices where ligands and proteins direct cell behaviours such as colonisation and proliferation, so called “third generation” biomaterials [33]. Beyond this third generation, biomaterials should encourage integration of man-made devices yet be able respond appropriately to biological or chemical cues should infection or rejection occur. For example pH responsive materials have been demonstrated to self-clean in response to local pH reductions due to bacteria colonisation [31]. For greater integration, enzymatic or other cell responses could be used to direct the behaviour of the material.

Magennis et al. recently showed a ‘bio-inspired’ approach in which bacterial enzymes mediate polymer synthesis at a cell surface and produces materials more favourable to cell binding than those synthesised in the absence of bacteria [83]. In this work it was demonstrated that polymers with co-monomer incorporation dependent on the templating bacterial species could be formed through polymerisation in the presence of cells. Utilising changes in redox potentials as a result of cell enzyme cascades combined with copper-mediated radical polymerisation and “click” chemistry, polymers with specific affinity to the template were generated *in situ.* Furthermore, fluorescent tagging of the polymers could take place at the bacterial surfaces directed by the same enzymes. This work showed how in complex biological systems, living processes and cell metabolism can be utilised to direct material properties. Combining these kinds of cellular biological pathways with third generation biomaterials and integrating their development into high-throughput screening approaches may lead to biomedical devices with much-improved functionalities for the future.

In summary, high-throughput strategies are leading to novel materials discovery when large numbers can be screened and “hits” identified retrospectively rather than planning those to yield positive results. Looking forward, despite the advantages of high-throughput methods, due to the vast variety of polymers which could potentially be synthesised, it is anticipated that we will see the increasing prominence of computation guided screening campaigns where initial descriptive results are then modelled and theoretical hits generated from virtual libraries. This approach will help to facilitate the next generation of highly functional polymer materials for safe, fully integrated biomedical devices and technologies.

## Disclosures

The authors have no conflict of interest to declare.

## References

[1] J. O’Neill, Review on Antimicrobial Resistance. Tackling a Crisis for the Health and Wealth of Nations, Commissioned by the UK Prime Minister, December 11, 2014.

[2] Patel I., Seemungal T., Wilks M., Lloyd-Owen S., Donaldson G., Wedzicha J. (2002). Relationship between bacterial colonisation and the frequency, character, and severity of COPD exacerbations. Thorax.

[3] Lockhart P.B., Brennan M.T., Sasser H.C., Fox P.C., Paster B.J., Bahrani-Mougeot F.K. (2008). Bacteremia associated with toothbrushing and dental extraction. Circulation.

[4] Roberts J., Sockett P. (1994). The socio-economic impact of human Salmonella enteritidis infection. Int. J. Food Microbiol..

[5] Fonkwo P.N. (2008). Pricing infectious disease: the economic and health implications of infectious diseases. EMBO Rep..

[6] McGlone S.M., Bailey R.R., Zimmer S.M., Popovich M.J., Tian Y., Ufberg P., Muder R.R., Lee B.Y. (2012). The economic burden of *Clostridium difficile*. Clin. Microbiol. Infect..

[7] Stone P.W. (2009). Economic burden of healthcare-associated infections: an American perspective. Expert Rev. Pharmacoecon. Outcomes Res..

[8] Magill S.S., Edwards J.R., Bamberg W., Beldavs Z.G., Dumyati G., Kainer M.A., Lynfield R., Maloney M., McAllister-Hollod L., Nadle J., Ray S.M., Thompson D.L., Wilson L.E., Fridkin S.K. (2014). Multistate point-prevalence survey of health care-associated infections. N. Engl. J. Med..

[9] Grainger D.W., van der Mei H.C., Jutte P.C., van den Dungen J.J.A.M., Schultz M.J., van der Laan B.F.A.M., Zaat S.A.J., Busscher H.J. (2013). Critical factors in the translation of improved antimicrobial strategies for medical implants and devices. Biomaterials.

[10] Belas R. (2014). Biofilms, flagella, and mechanosensing of surfaces by bacteria. Trends Microbiol..

[11] Costerton J.W., Stewart P.S., Greenberg E.P. (1999). Bacterial biofilms: a common cause of persistent infections. Science.

[12] Smith A.W. (2005). Biofilms and antibiotic therapy: is there a role for combating bacterial resistance by the use of novel drug delivery systems?. Adv. Drug Deliv. Rev..

[13] Donlan R.M. (2001). Biofilms and device-associated infections. Emerg. Infect. Dis..

[14] Mah T., O’Toole G. (2001). Mechanisms of biofilm resistance to antimicrobial agents. Trends Microbiol..

[15] Anwar H., Strap J.L., Chen K., Costerton J.W. (1992). Dynamic interactions of biofilms of mucoid *Pseudomonas aeruginosa* with tobramycin and piperacillin. Antimicrob. Agents Chemother..

[16] Evans R.C., Holmes C.J. (1987). Effect of vancomycin hydrochloride on *Staphylococcus epidermidis* biofilm associated with silicone elastomer. Antimicrob. Agents Chemother..

[17] Kollef M.H., Fraser V.J. (2001). Antibiotic resistance in the intensive care unit. Ann. Intern. Med..

[18] Timofeeva L., Kleshcheva N. (2011). Antimicrobial polymers: mechanism of action, factors of activity, and applications. Appl. Microbiol. Biotechnol..

[19] Siedenbiedel F., Tiller J.C. (2012). Antimicrobial polymers in solution and on surfaces: overview and functional principles. Polymers.

[20] Kuroda K., Caputo G.A. (2013). Antimicrobial polymers as synthetic mimics of host-defense peptides. Wiley Interdiscip. Rev. Nanomed. Nanobiotechnol..

[21] Murata H., Koepsel R.R., Matyjaszewski K., Russell A.J. (2007). Permanent, non-leaching antibacterial surfaces—2: how high density cationic surfaces kill bacterial cells. Biomaterials.

[22] Chen S., Li L., Zhao C., Zheng J. (2010). Surface hydration: principles and applications toward low-fouling/nonfouling biomaterials. Polymer.

[23] Kane R.S., Deschatelets P., Whitesides G.M. (2003). Kosmotropes form the basis of protein-resistant surfaces. Langmuir.

[24] Merrill E., Harris J.M. (1992). Poly(ethylene oxide) and blood contact. Poly(Ethylene Glycol) Chemistry.

[25] M. Milan, M.W. George, Using Self-Assembled Monolayers That Present Oligo(ethylene glycol) Groups To Control the Interactions of Proteins with Surfaces. Poly(ethylene glycol), American Chemical Society, 1997, pp. 361–373.

[26] Kingshott P., Wei J., Bagge-Ravn D., Gadegaard N., Gram L. (2003). Covalent attachment of poly(ethylene glycol) to surfaces, critical for reducing bacterial adhesion. Langmuir.

[27] Prime K.L., Whitesides G.M. (1993). Adsorption of proteins onto surfaces containing end-attached oligo(ethylene oxide): a model system using self-assembled monolayers. J. Am. Chem. Soc..

[28] Prime K.L., Whitesides G.M. (1991). Self-assembled organic monolayers: model systems for studying adsorption of proteins at surfaces. Science.

[29] Cunliffe D., Smart C.A., Alexander C., Vulfson E.N. (1999). Bacterial adhesion at synthetic surfaces. Appl. Environ. Microbiol..

[30] Sin M.-C., Chen S.-H., Chang Y. (2014). Hemocompatibility of zwitterionic interfaces and membranes. Polym. J..

[31] Gensel J., Borke T., Pérez N.P., Fery A., Andreeva D.V., Betthausen E., Müller A.H.E., Möhwald H., Skorb E.V. (2012). Cavitation engineered 3D sponge networks and their application in active surface construction. Adv. Mater..

[32] Van Loosdrecht M., Lyklema J., Norde W., Schraa G., Zehnder A. (1987). Electrophoretic mobility and hydrophobicity as a measured to predict the initial steps of bacterial adhesion. Appl. Environ. Microbiol..

[33] Hench L.L., Polak J.M. (2002). Third-generation biomedical materials. Science.

[34] Dou X.-Q., Zhang D., Feng C., Jiang L. (2015). Bioinspired hierarchical surface structures with tunable wettability for regulating bacteria adhesion. ACS Nano.

[35] Sanni O., Chang C.-Y., Anderson D.G., Langer R., Davies M.C., Williams P.M., Williams P., Alexander M.R., Hook A.L. (2014). Bacterial attachment to polymeric materials correlates with molecular flexibility and hydrophilicity. Adv. Healthcare Mater..

[36] Arciola C.R., Campoccia D., Speziale P., Montanaro L., Costerton J.W. (2012). Biofilm formation in Staphylococcus implant infections. A review of molecular mechanisms and implications for biofilm-resistant materials. Biomaterials.

[37] Ong Y.-L., Razatos A., Georgiou G., Sharma M.M. (1999). Adhesion forces between *E. coli* bacteria and biomaterial surfaces. Langmuir.

[38] Smith R.S., Zhang Z., Bouchard M., Li J., Lapp H.S., Brotske G.R., Lucchino D.L., Weaver D., Roth L.A., Coury A., Biggerstaff J., Sukavaneshvar S., Langer R., Loose C. (2012). Vascular catheters with a nonleaching poly-sulfobetaine surface modification reduce thrombus formation and microbial attachment. Sci. Transl. Med..

[39] Biointeractions, Avert – Surface Active Antimicrobial Coating Technical Specification, <www.biointeractions.com> 2015, Technical Specification.

[40] Medtronic. TYRX™Product overview, <www.tyrx.com> 2015.

[41] Shariff N., Eby E., Adelstein E., Jain S., Shalaby A., Saba S., Wang N.C., Schwartzman D. (2015). Health and economic outcomes associated with use of an antimicrobial envelope as a standard of care for cardiac implantable electronic device implantation. J. Cardiovasc. Electrophysiol..

[42] Gristina A.G. (1987). Biomaterial-centered infection: microbial adhesion versus tissue integration. Science.

[43] Busscher H.J., van der Mei H.C., Subbiahdoss G., Jutte P.C., van den Dungen J.J.A.M., Zaat S.A.J., Schultz M.J., Grainger D.W. (2012). Biomaterial-associated infection: locating the finish line in the race for the surface. Sci. Transl. Med..

[44] Ratner B.D., Bryant S.J. (2004). Biomaterials: where we have been and where we are going. Annu. Rev. Biomed. Eng..

[45] Mei Y., Saha K., Bogatyrev S.R., Yang J., Hook A.L., Kalcioglu I., Cho S.-W., Mitalipova M., Pyzocha N., Rojas F., Vliet K.J.V., Davies M.C., Alexander M.R., Langer R., Jaenisch R., Anderson D.G. (2010). Combinatorial development of biomaterials for clonal growth of human pluripotent stem cells. Nat. Mater..

[46] A. Celiz, Cell Culture Substrate, UK Patent Application, 2014.

[47] Celiz A., Smith J., Langer R., Anderson D., Winkler D.A., Epa V.C., Barrett D., Young L., Denning C., Davies M., Alexander M. (2014). Materials for stem cell factories of the future. Nat. Mater..

[48] Hook A.L., Chang C.Y., Yang J., Atkinson S., Langer R., Anderson D.G., Davies M.C., Williams P., Alexander M.R. (2013). Discovery of novel materials with broad resistance to bacterial attachment using combinatorial polymer microarrays. Adv. Mater..

[49] Hook A.L., Chang C.-Y., Yang J., Luckett J., Cockayne A., Atkinson S., Mei Y., Bayston R., Irvine D.J., Langer R., Anderson D.G., Williams P., Davies M.C., Alexander M.R. (2012). Combinatorial discovery of polymers resistant to bacterial attachment. Nat. Biotechnol..

[50] Engler A., Sen S., Sweeney H., Discher D. (2006). Matrix elasticity directs stem cell lineage specification. Cell.

[51] Dalby M.J., Gadegaard N., Tare R., Andar A., Riehle M.O., Herzyk P., Wilkinson C.D.W., Oreffo R.O.C. (2007). The control of human mesenchymal cell differentiation using nanoscale symmetry and disorder. Nat. Mater..

[52] Benoit D., Schwartz M., Durney A., Anseth K. (2008). Small functional groups for controlled differentiation of hydrogel-encapsulated human mesenchymal stem cells. Nat. Mater..

[53] Kohn J. (2004). New approaches to biomaterials design. Nat. Mater..

[54] Kell D., Oliver S. (2004). Here is the evidence, now what is the hypothesis? The complementary roles of inductive and hypothesis-driven science in the post-genomic era. Bioessays.

[55] Anderson D.G., Levenberg S., Langer R. (2004). Nanoliter-scale synthesis of arrayed biomaterials and application to human embryonic stem cells. Nat. Biotechnol..

[56] H. Mizomoto, The synthesis and screening of polymer libraries using a high throughput approach (Southampton Ph.D. thesis), University of Southampton, 2004.

[57] Urquhart A.J., Taylor M., Anderson D.G., Langer R., Davies M.C., Alexander M.R. (2008). TOF-SIMS analysis of a 576 micropatterned copolymer array to reveal surface moieties that control wettability. Anal. Chem..

[58] Urquhart A.J., Anderson D.G., Taylor M., Alexander M.R., Langer R., Davies M.C. (2007). High throughput surface characterisation of a combinatorial material library. Adv. Mater..

[59] Epa V.C., Hook A.L., Chang C., Yang J., Langer R., Anderson D.G., Williams P., Davies M.C., Alexander M.R., Winkler D.A. (2014). Modelling and prediction of bacterial attachment to polymers. Adv. Funct. Mater..

[60] Epa V.C., Yang J., Mei Y., Hook A.L., Langer R., Anderson D.G., Davies M.C., Alexander M.R., Winkler D.A. (2012). Modelling human embryoid body cell adhesion to a combinatorial library of polymer surfaces. J. Mater. Chem..

[61] Cranford S.W., de Boer J., van Blitterswijk C., Buehler M.J. (2013). Materiomics: an-omics approach to biomaterials research. Adv. Mater..

[65] Hori K., Matsumoto S. (2010). Bacterial adhesion: from mechanism to control. Biochem. Eng. J..

[69] Brocchini S., James K., Tangpasuthadol V., Kohn J. (1998). Structure–property correlations in a combinatorial library of degradable biomaterials. J. Biomed. Mater. Res..

[70] Hook A.L., Anderson D.G., Langer R., Williams P., Davies M.C., Alexander M.R. (2010). High throughput methods applied in biomaterial development and discovery. Biomaterials.

[71] Hook, Novel polymers which resist bacterial attachment UK Patent Application no: 1107416.8, 2011.

[72] Pernagallo S., Wu M., Gallagher M.P., Bradley M. (2011). Colonising new frontiers—microarrays reveal biofilm modulating polymers. J. Mater. Chem..

[73] Venkateswaran S., Wu M., Gwynne P.J., Hardman A., Lilienkampf A., Pernagallo S., Blakely G., Swann D.G., Gallagher M.P., Bradley M. (2014). Bacteria repelling poly(methylmethacrylate-co-dimethylacrylamide) coatings for biomedical devices. J. Mater. Chem. B.

[74] Hook A.L., Yang J., Chen X., Roberts C.J., Mei Y., Anderson D.G., Langer R., Alexander M.R., Davies M.C. (2011). Polymers with hydro-responsive topography identified using high throughput AFM of an acrylate microarray. Soft Matter.

[75] Taylor M., Urquhart A.J., Zelzer M., Davies M.C., Alexander M.R. (2007). Picoliter water contact angle measurement on polymers. Langmuir.

[76] Hook A.L., Thissen H., Voelcker N.H. (2009). Surface plasmon resonance imaging of polymer microarrays to study protein−polymer interactions in high throughput. Langmuir.

[77] Davies M.C., Alexander M.R., Hook A.L., Yang J., Mei Y., Taylor M., Urquhart A.J., Langer R., Anderson D.G. (2010). High throughput surface characterization: a review of a new tool for screening prospective biomedical material arrays. J. Drug Target..

[78] Taylor M., Urquhart A.J., Anderson D.G., Langer R., Davies M.C., Alexander M.R. (2009). Partial least squares regression as a powerful tool for investigating large combinatorial polymer libraries. Surf. Interface Anal..

[79] Hook A.L., Alexander M.R., Winkler D.A., Blitterswijk C.A.V., Boer J.D. (2014). Chapter 8 – Materiomics: a toolkit for developing new biomaterials. Tissue Engineering.

[80] Cranford S.W., Buehler Markus J. (2012). Biomateriomics.

[81] Katritzky A.R., Lobanov V.S., Karelson M. (1995). QSPR: the correlation and quantitative prediction of chemical and physical properties from structure. Chem. Soc. Rev..

[82] Le T., Epa V.C., Burden F.R., Winkler D.A. (2012). Quantitative structure–property relationship modeling of diverse materials properties. Chem. Rev..

[83] Magennis E.P., Fernandez-Trillo F., Sui C., Spain S.G., Bradshaw D.J., Churchley D., Mantovani G., Winzer K., Alexander C. (2014). Bacteria-instructed synthesis of polymers for self-selective microbial binding and labelling. Nat. Mater..

